# Plantar Fasciitis: An Autobiographical Case Report From the Perspective of an Athlete and Physician

**DOI:** 10.7759/cureus.81623

**Published:** 2025-04-02

**Authors:** Nicholas C Watson

**Affiliations:** 1 Critical Care, Anesthesia Practice Consultants, Grand Rapids, USA

**Keywords:** autobiographical case report, physical therapy modalities, plantar fasciitis, running, sports medicine, supershoes

## Abstract

Plantar fasciitis (PF) is a common cause of foot pain. Individual therapies lack substantial evidence to identify a reliable treatment algorithm, but several modalities have been associated with clinical improvement. This case report reviews the experience of the author, an endurance athlete and physician, who applied various interventions over the course of two years. The goal is to exemplify the challenges typical of PF and offer perspective on solutions that were trialed based on published evidence and systematic application of first principles. Counterintuitive and efficacious therapies are highlighted.

## Introduction

Plantar fasciitis (PF) is a common condition presenting as pain at the calcaneal anteromedial prominence. PF accounts for more than one million annual visits to physicians in the USA, with an incidence in runners of 4.5%-10% [1,2]. The proposed mechanism of PF is biomechanical overuse resulting in degenerative changes in the plantar fascia attachment to the calcaneus, although the exact mechanisms are not well understood [2]. The histologic absence of inflammation indicates that the disease is more properly termed degenerative plantar fasciosis [3]. PF can typically be diagnosed clinically based on history and physical examination [4,5]. Patients commonly report pain with the first step in the morning, a gradual reduction of pain with activity, and return of pain during ambulation after a period of inactivity. Pain with palpation at the anteromedial calcaneus and similar pain with forced dorsiflexion of the toes at the metatarsophalangeal joints, with the ankle stabilized, reliably identifies PF [4,5]. 

The majority of patients with PF improve within a year with nonoperative management. Individual therapies lack substantial evidence to identify a reliable treatment algorithm, but many modalities have been associated with clinical improvement [2,4-6]. Tailoring therapy to individual patient characteristics and response is supported in medical literature and anecdotally in internet forums [4,6,7].

This case report highlights the experience of the author, an accomplished endurance athlete and physician, who trialed various interventions over the course of two years. The goal is to exemplify the challenges typical of PF and offer perspective on solutions that were trialed based on published evidence and the systematic application of the fundamental principles for the treatment of a running-related injury.

## Case presentation

I am a 47-year-old man who has been an endurance runner since age 13. My athletic pursuits include endurance running, long-distance triathlon (swim-bike-run), and duathlon (run-bike-run). I have run approximately 112,000 kilometers in an athletic career marked by a paucity of injuries.

In 2019, at the age of 44 years, I was 180.3 cm in height and 65 kg in body mass. My estimated running maximal oxygen consumption (VO2 max) was 61 ml/kg/min based on 16:43 for a recent 5,000 m race [8]. My average weekly training time was 12-15 hours. The weekly training load was split between 4-6 hours of running and 8-10 hours of bicycling, divided into approximately 80% maintenance (easy) and 20% higher intensity (lactate threshold and greater) [9]. As has been my lifetime practice, I did maintenance runs in my usual training shoes and did high-intensity running workouts in my racing shoes in order to be fully acclimatized to their unique qualities. 

My racing shoes for 2019 were a pair of “supershoes,” defined here as any running shoe comprised of high-energy return foam and a carbon-plated sole [10,11]. At the time, such shoes were relatively new to the market, and this was my first experience with them. Peer-reviewed scientific inquiry has shown that supershoes improve running economy, that is, they reduce the oxygen requirement for a given running pace [11]. In short, most runners enjoy a performance benefit when using supershoes. My experience showed a consistent 1%-2% improvement in running speed across all paces with supershoes. After my first run in the supershoes, the soles of my feet were diffusely sore, an uncommon experience for me. Over the next few months I continued to run in the supershoes once or twice per week, having similar (yet tolerable) discomfort for a few days after each workout. All other runs were done in my usual training shoes, which initially did not exacerbate the discomfort. Downhill running in the supershoes elicited significant pain in my heels and a tight sensation across the arch of my foot. Of note, after a hilly and particularly long race, my medial ankles and soles were very painful, 8 out of 10 on the visual analog scale, with 2+ pitting edema. The acute pain increased slightly with each run, and chronic pain accumulated at an almost imperceptible rate. Nine months after I first ran in the supershoes, I had daily rest pain in the soles of both feet, tenderness to palpation at the anteromedial calcaneus, and daily morning first-step pain, and I was unable to tolerate running in any shoes on any surface. My sports medicine physician made the diagnosis of PF based on history and physical exam.

Rest

Common sense suggests that if running is painful, then abstaining from running may be beneficial, especially in the context of presumed tissue damage. Rest may allow time for microtrauma to heal. Rest should also allow any inflammation to subside, although PF is histologically a degenerative fasciosis rather than inflammation. I initially abstained from all exercise for 14 days with no noticeable improvement in rest pain or standing pain. Next, I decided to bicycle daily, which tended to cause little to no pain during the activity and did not affect rest pain or standing pain after exercise. Notably, my bicycling shoes have an extremely stiff carbon foot plate and arch support that minimize pronation and eliminate the heel-to-toe rocking motion associated with heel-strike running. The first run after this rest period was excruciating. I gradually reintroduced running (training shoes only) one or two times per week for 10-20 minutes per run. Running remained acutely painful, and chronic pain was unremitting for four months with this approach of greatly reduced running volume. In general, rest had no influence on acute or chronic plantar pain.

Pharmaceuticals

Steroid injection into the plantar fascia may reduce pain acutely but has not been shown to consistently provide better long-term pain control than other approaches [2,5]. Because steroid injection is associated with a risk of plantar fascia rupture and fat pad atrophy, I chose to forgo this option. While abstaining from running, I also took scheduled acetaminophen and ibuprofen for 14 days. Similar to steroids, the anti-inflammatory effect of ibuprofen may be beneficial, although there is no histological evidence of tissue inflammation [3,4]. The analgesic effect was modest and transient, improving my tolerance for walking and standing at work. When I returned to running, there was no apparent change in pain. Moreover, acetaminophen and ibuprofen taken before and after running had no appreciable effect on acute or chronic pain.

Shoes

**Figure 1:**
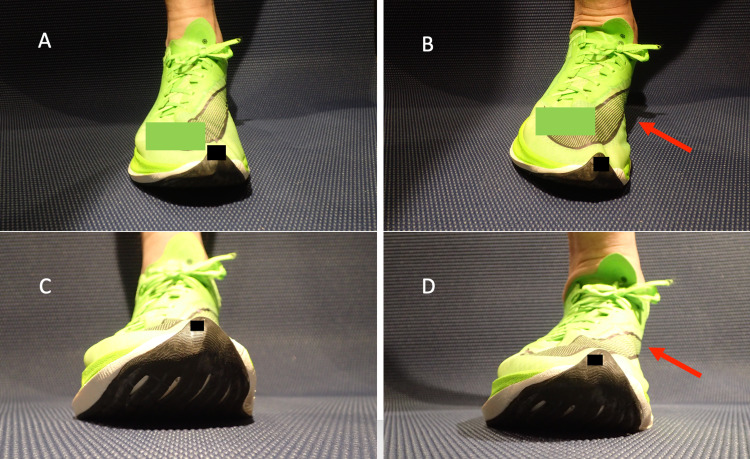
Pronation in supershoes A: Midstance with neutral position, 30° view.  B: Toe-off with pronation, 30° view.  C: Midstance with neutral position, 0° view.  D: Toe-off with pronation, 0° view.  Red arrows identify overpronation

Running in supershoes caused an exaggerated pronation with footstrike compared to running in training shoes (Figure 1). 

Voluntarily forcing my foot into overpronation while bearing body weight with or without shoes reproduced the plantar pain, leading me to believe that at least some of the mechanisms of pain were related to pronation during running in the supershoes. Additionally, the drop (change in sole height from heel to toe) in the supershoes was 10 mm, greater than any training shoes I used. 

**Figure 2:**
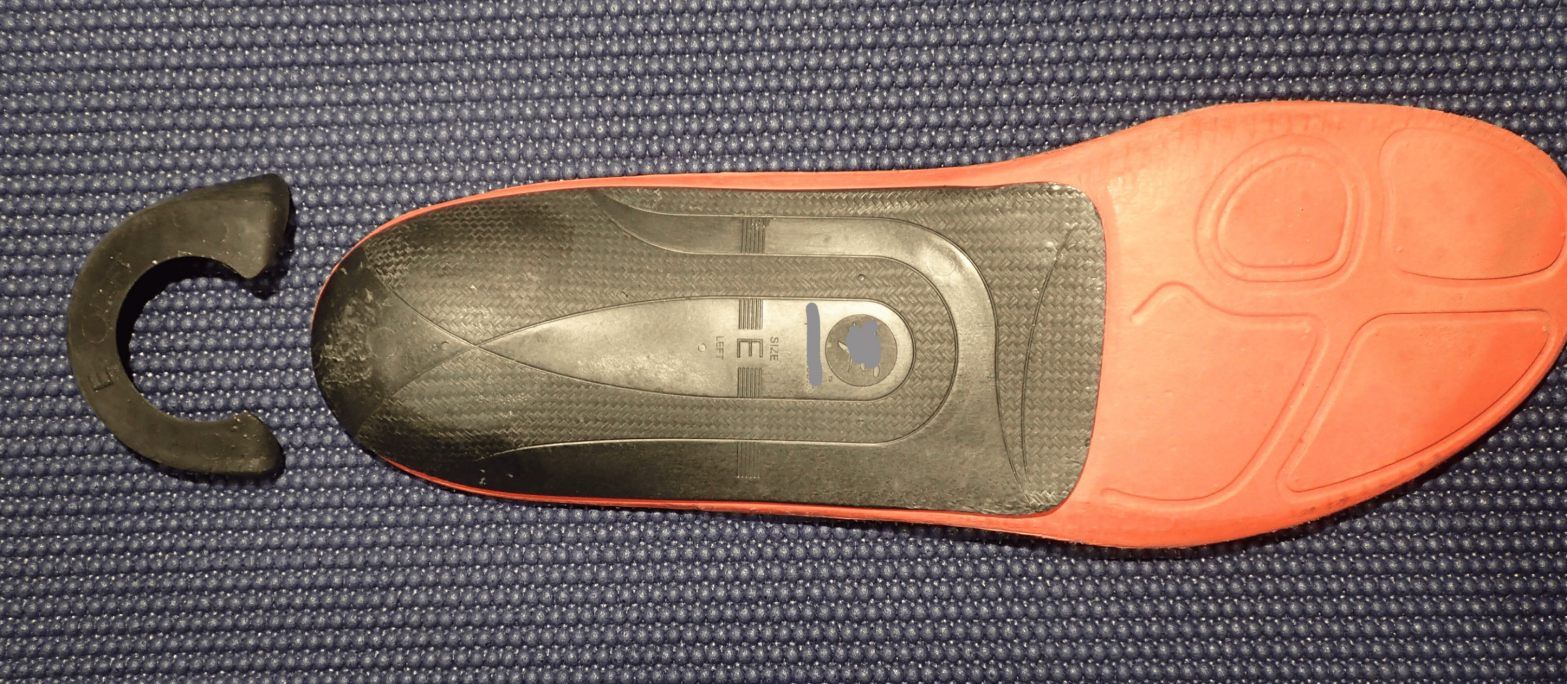
Shoe insert with heel stabilizer Heel stabilizer (left) and orthotic (right)

**Figure 3:**
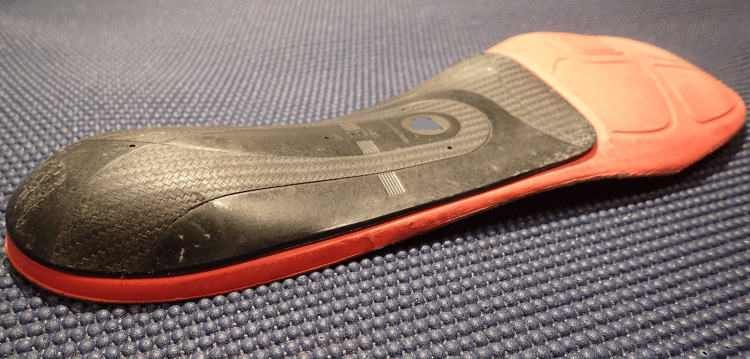
Shoe insert Acute angle perspective to highlight the heel cup

I underwent a series of deliberate trials aimed at addressing the mechanics of the supershoes that theoretically provoked the PF. I reasoned that running with more arch support or other features that resisted extreme pronation would be beneficial, a notion supported by the absence of pain when wearing bicycling shoes with a stiff footplate. It was unclear to me whether the drop of the shoe had any effect. First, I stopped running in supershoes. I deliberately experimented with multiple training shoes manufactured by several companies and was unable to find a pair that provided relief while running or standing. I tried shoes designed for runners who “overpronate,” shoes made for stability, zero- and low-drop shoes, minimalist shoes, and maximalist shoes. The first change that resulted in a noticeable improvement was a hard plastic shoe insert that matched the contour of my arches and formed a cup shape around my heel (Figures 2, 3). This device significantly reduced pain during and after running, regardless of the training shoes it was paired with. I also adopted the use of this insert in my daily work shoes with a marked and sustained relief in daily standing pain, rest pain, and morning standing pain. 

**Figure 4:**
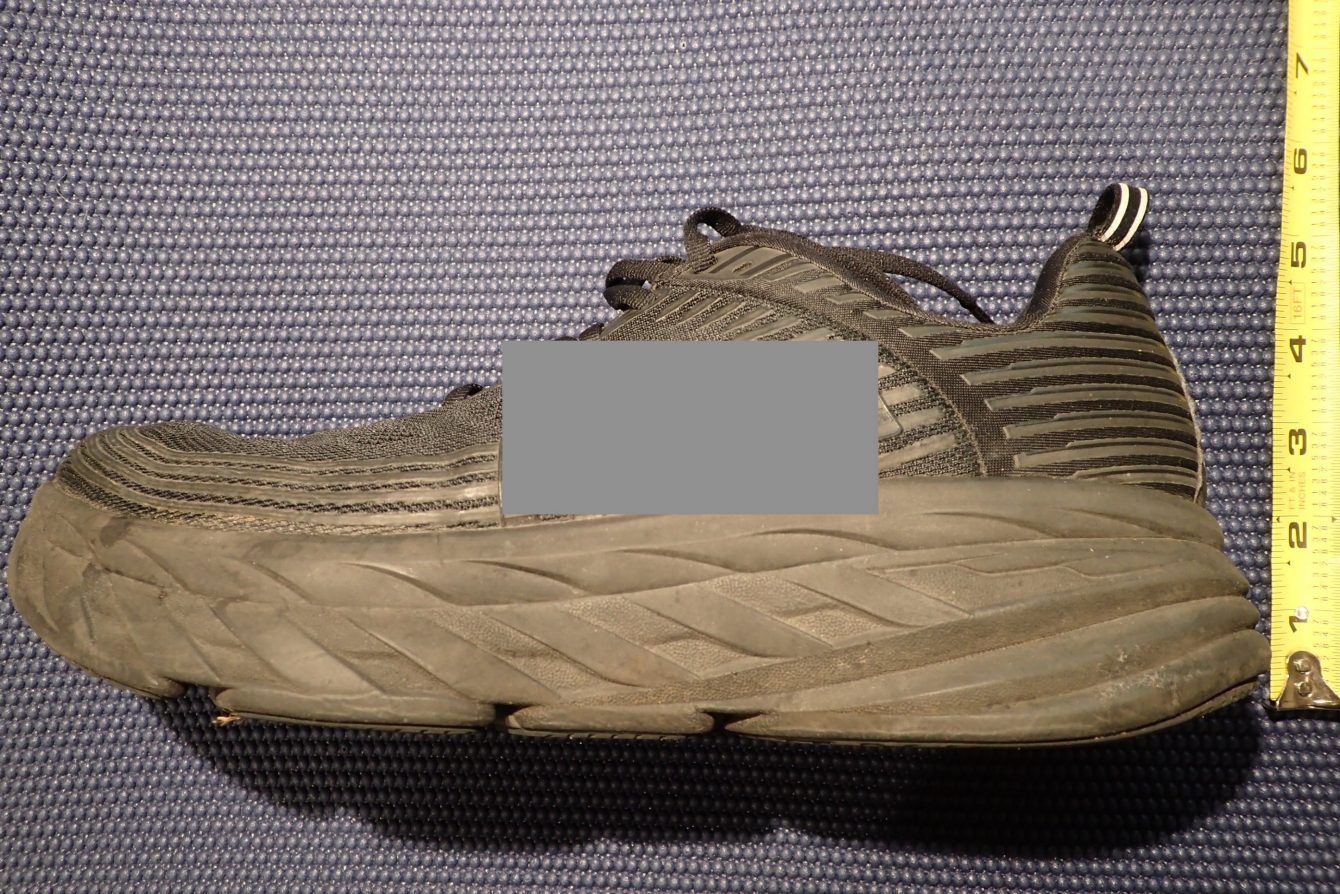
Example of a maximalist running shoe Ruler (inches) shown to identify the deep cushion

For six months, I trained in maximalist shoes with a 4 mm drop (Figure 4) with the hard plastic insert, achieving a level of acceptable pain during and after running that allowed me to train consistently at my preinjury volumes and intensities. I then transitioned to lighter and more flexible training shoes (also 4 mm drop) combined with the hard plastic insert, settling into a level of acceptable pain at 3 out of 10 during and after running. I continued to have moderate chronic rest pain and morning standing pain.

Stretching and physical therapy

Stretching and strengthening may reduce pain from PF [4,5]. Shortly after being formally diagnosed with PF, I began a physical therapy regimen. A physical therapist identified various opportunities for improvement concerning the flexibility and strength of my spine, hips, knees, and ankles. I faithfully followed the therapy prescriptions I was given, amounting to 30 minutes of dedicated therapy per day for three months. The cumulative effects of physical therapy were increased global flexibility and strength, but one element was distinctly beneficial. Eventually, I felt that the most useful of these exercises for reducing plantar pain was maximal ankle dorsiflexion with the knee flexed. This involved unilateral kneeling (genuflexion) with the contralateral foot flat on the floor and torso weight pressed into the knee to load the ankle in dorsiflexion. This stretch, when held for 30 seconds and repeated three times, followed the same pattern of feedback for months: extreme pain in the plantar aspect of the foot on the first bout, with pain reducing to zero by the third bout and remaining minimal for a few hours poststretch. Each successive bout resulted in a greater degree of dorsiflexion. I used this stretch for acute pain relief multiple times per day, including when first walking in the morning, before/during/after running, and during routine work days. Even after improvement in chronic pain, I continue to perform this stretch on a daily basis with prompt relief of acute pain.

Moreover, I also wore a plantar boot for passive stretching. This device is a boot with straps designed for pulling the ankle into dorsiflexion. I tolerated sleeping with this for four weeks, experiencing numbness in my toes and feet. Because it was very difficult to sleep while wearing the boot, I aimed instead for 30 minutes per day of cumulative boot use while awake. This approach did reduce the pain after the stretch, but it was so painful during implementation that I eventually discontinued its use.

I also tried a number of other interventions designed to physically modify soft tissue. Rolling a soft or hard ball under foot, instrument-assisted soft tissue mobilization, massage, vibration, heat, and cold all provided a low level of transient pain relief but were otherwise unimpressive and therefore abandoned.

Personal guidelines

Approximately six months after the diagnosis of PF, I had used trial-and-error to develop a set of personal guidelines for minimizing pain related to running and in daily nonathletic life. Each of these practices was based on the simple principle that any factor reducing plantar pain acutely would eventually lead to chronic pain reduction and ultimately resolution. I wore foam slippers with high arch support rather than walking barefoot in my home. My daily shoes were maximalist with the hard plastic insert. I ran exclusively in training shoes with hard plastic inserts and a broad base that limited pronation. I avoided slanted running surfaces such as crowned roads or canted trails to avoid excessive pronation. I also limited downhill running as this continued to be a source of irritation. Each run started with severe pain that would improve over 10 minutes, so I adopted a strategy of warming up with a slow run and deep dorsiflexion stretching before getting up to my normal run speeds. I continued with the physical therapy regimen on a daily basis.

A year after the original PF diagnosis, I had only mild chronic pain with occasional acute exacerbations. Over the next year (13-24 months after the original diagnosis), I continued to apply the personal guidelines and the pain faded very slowly, eventually reaching a point where the chronic pain was gone, running was painless, and minimal pain was only noticeable the day after a very long or very intense running workout. During this span of time, I had periods where I would reduce running frequency and/or running volume due to precompetition taper, postcompetition rest, or a focused bicycling-specific training block. Each time I reduced running frequency or volume, the acute and chronic pain would return. This was most extreme when I took a 10-day break from running after a competition season. In this situation, I had severe acute pain that limited running for a week, and the daily rest pain took four weeks to resolve with routine running. It was at this point that I added the last item to my personal guidelines: avoid prolonged abstinence from running.

**Figure 5:**
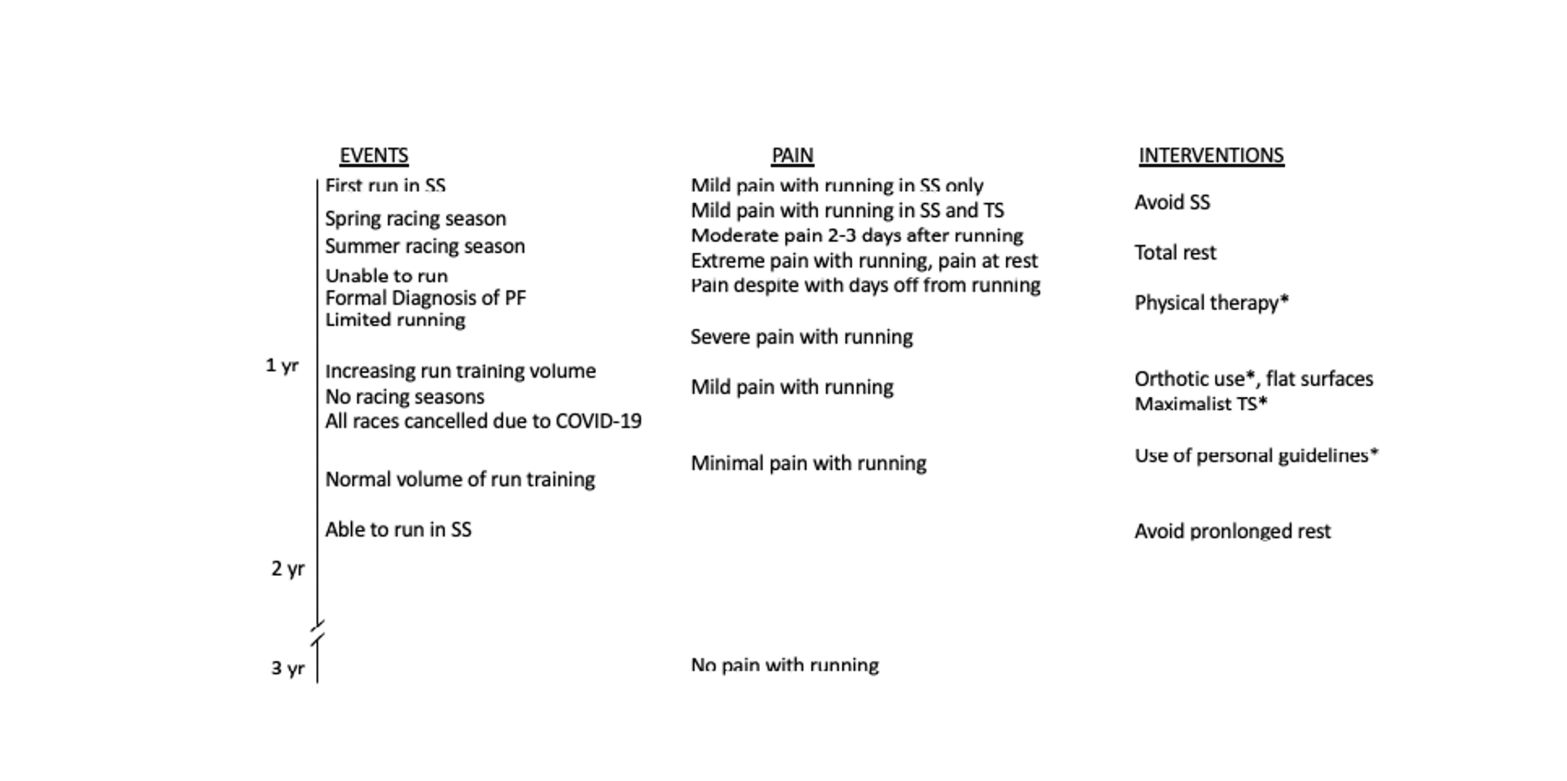
Timeline of events SS: super shoes; TS: training shoes; yr: year; PF: plantar fasciitis *Intervention was continued

Almost exactly two years from the time of the PF diagnosis, with the routine application of my personal guidelines, I was able to run pain-free, and this has remained the case for years (Figure 5). Deviation from these guidelines is typically followed by a few days of rest pain and pain with running. I can train and race in supershoes without a special insert and have virtually no limitation on running volume or intensity. My running fitness has also returned to preinjury levels.

## Discussion

The literature pertaining to PF identifies multiple available therapies, but interventions that reliably reduce or eliminate PF pain remain elusive [2,4-6]. Searching internet sports forums for “plantar fascia” makes abundantly clear that PF has no ubiquitous solution [7]. Indeed, it appears that patient response to intervention is highly variable: what works well for one person may fail entirely for another. A common theme among online reports is trialing multiple simultaneous therapies until improvement is noted, with a very wide range of time to improvement (e.g., weeks to years). In particular, runners tend to alter factors associated with running itself (e.g., shoes, terrain, training volume/intensity) and measure the efficacy of their therapies based on the ability to return to normal training patterns [7]. This case report demonstrates exactly this approach based on published evidence and first principles.

Rest (abstaining from running) would be expected to reduce PF pain and is typically an early intervention for most runners in this circumstance. Interestingly, I wish to highlight in this case report that rest and anti-inflammatory medications had virtually no benefit. Similarly, a prolonged period of reduced running volume and intensity had little noticeable effect on acute and chronic pain. Once I was able to train routinely, the next discovery was that the acute and chronic PF pain would return if I went several days without running! While this contradicts standard wisdom for running injuries in general, this phenomenon is not without precedent. As Matt Fitzgerald has described in his own counterintuitive experience with PF, “So an injury that was caused by running and perhaps exacerbated by rest was ultimately cured by running” [12].

This case report is limited in that not all published therapies were available or trialed. It is impossible to know if any of the therapies contributed to overall improvement in PF aside from small daily changes in acute pain that were temporally associated with the interventions. The possibility of natural progression and spontaneous resolution cannot be eliminated.

## Conclusions

PF can be very difficult to treat, and the indolent course is a constant source of frustration. A sensible approach is to seek professional guidance while also systematically pursuing therapies based on published evidence and first principles. A comprehensive approach should consider the counterintuitive possibility that consistent running stress may contribute to improvement. This case report highlights the individualistic nature and trial-and-error process of PF management.
